# Sicily statement on evidence-based practice

**DOI:** 10.1186/1472-6920-5-1

**Published:** 2005-01-05

**Authors:** Martin Dawes, William Summerskill, Paul Glasziou, Antonino Cartabellotta, Janet Martin, Kevork Hopayian, Franz Porzsolt, Amanda Burls, James Osborne

**Affiliations:** 1 Department of Family Medicine. McGill University, Montreal, Canada; 2 The Lancet, Jamestown Road, London, UK; 3 Department of Primary Health Care, Centre for Evidence-Based Practice, Oxford University, Oxford, UK; 4 Gruppo Italiano per la Medicina Basata sulle Evidenze (GIMBE), Passaggio L. da Vinci, 16 – 90145 Palermo, Italy; 5 London Health Sciences Centre, Department of Physiology & Pharmacology, University of Western Ontario, London, Ontario, Canada; 6 School of Medicine, Health Policy and Practice, University of East Anglia Norwich, UK; 7 University Hospital Ulm, Clinical Economics, Ulm, Germany; 8 Department Public Health and Epidemiology, University of Birmingham, Birmingham UK; 9 United Bristol Healthcare Trust, Bristol, UK

## Abstract

**Background:**

A variety of definitions of evidence-based practice (EBP) exist. However, definitions are in themselves insufficient to explain the underlying *processes *of EBP and to differentiate between an evidence-based process and evidence-based *outcome*. There is a need for a clear statement of what Evidence-Based Practice (EBP) means, a description of the skills required to practise in an evidence-based manner and a curriculum that outlines the minimum requirements for training health professionals in EBP. This consensus statement is based on current literature and incorporating the experience of delegates attending the 2003 Conference of Evidence-Based Health Care Teachers and Developers ("Signposting the future of EBHC").

**Discussion:**

Evidence-Based Practice has evolved in both scope and definition. Evidence-Based Practice (EBP) requires that decisions about health care are based on the best available, current, valid and relevant evidence. These decisions should be made by those receiving care, informed by the tacit and explicit knowledge of those providing care, within the context of available resources.

Health care professionals must be able to gain, assess, apply and integrate new knowledge and have the ability to adapt to changing circumstances throughout their professional life. Curricula to deliver these aptitudes need to be grounded in the five-step model of EBP, and informed by ongoing research. Core assessment tools for each of the steps should continue to be developed, validated, and made freely available.

**Summary:**

All health care professionals need to understand the principles of EBP, recognise EBP in action, implement evidence-based policies, and have a critical attitude to their own practice and to evidence. Without these skills, professionals and organisations will find it difficult to provide 'best practice'.

## Background

### The Sicily statement on evidence-based practice

"Knowing is not enough; we must apply. Willing is not enough, we must do" [1]

Health care delivered in ignorance of available research evidence, misses important opportunities to benefit patients and may cause significant harm [2-4]. Providing evidence-based care is recognised as a key skill for health care workers from diverse professions and cultures [5-10]. The ability to deliver evidence-based practice promotes individualisation of care and assures the quality of health care for patients today as well as those of tomorrow [11].

A variety of definitions of evidence-based practice (EBP) have been proposed. However, definitions are in themselves insufficient to explain the underlying *processes *of EBP and to differentiate between an evidence-based process and evidence-based *outcome*.

Towards this goal, we propose three points to clarify and promote the realisation of EBP:

1) A clear statement of what EBP means.

2) A description of the minimum skill set required to practise in an evidence-based way.

3) A curriculum that outlines the minimum standard educational requirements for training health professionals in EBP.

This statement was conceived by the delegates of the second international conference of Evidence-Based Health Care Teachers and Developers held in Sicily in September 2003 ("Signposting the future of EBHC", [12]). In response to a request from the delegates at this conference's final plenary session the steering committee prepared the first draft. The proposed statement and a topic questionnaire were then circulated to all 86 attendees of the Sicily conference for suggestions and clarifications. Eighteen professions allied to health from 18 countries were represented. Suggestions were incorporated and a final paper approved by consensus.

## Discussion

### Increase in medical information

During the last century there has been an exponential growth of research and knowledge [13,14]. The growth of health care information has been particularly rapid in diagnostic and therapeutic technologies. The volume of medical papers published doubles every 10 to 15 years [15]. Electronic searching of this expanding evidence base was initiated by the National Library of Medicine in 1966 [16]. Electronic access to full text articles and journals started to become available in 1998 [17]. Increasingly, specialist databases of utility for health professionals are being developed, such as the Physiotherapy Evidence Database [18] and the C2-SPECTR [19]. Regular use of these resources is identified as one marker for lifelong learning among physicians [20], but the process is not easy [21]. Identification of the best methods to understand and integrate patient values, such as decision aids or patient-centred consultations, is still at the early stages of development [22].

With this expansion of information, our knowledge should be greater and our practice should be more effective. Unfortunately this is too often not the case [23]. This recognised gap between best evidence and practice is one of the driving forces behind the development of EBP.

### Clinical decision making

Good practice including effective clinical decision making – step 4 of the EBP process – requires the explicit research evidence and non-research knowledge (tacit knowledge or accumulated wisdom). Clinical decision making is the end point of a process that includes clinical reasoning, problem solving, and awareness of patient and health care context [24]. This process is uncertain and frequently no "correct" decision exists. EBP can help with *some *of the uncertainties in this decision process by using the explicit knowledge obtainable from research information. But to do so the research information must be transformed into clinicians' knowledge. Information can be defined as data that has been sorted, analysed, & displayed and communicated through language, graphic displays, or numeric tables. Explicit knowledge is then the meaning people create using this information and its application through action in specific settings [25]. For example clinician's knowledge should include the need to evaluate quickly the patient with chest pain to take advantage of the research proven window of opportunity for treatment of acute coronary syndrome. Step 4 also requires the tacit knowledge which comes from the wisdom of experience, informed by evidence and outcomes, and which is consequently harder to share. An example is the recognition of a sick child. Research may develop a list of clinical features that, when present, denote severe illness in a child. While this list will help the inexperienced junior doctor, nurse, or midwife, the experienced health practitioner has a tacit knowledge of "sickness" in a child that comes from both knowledge of the features list and assimilation with experience, thereby speeding up the recognition of "sickness" in a child.

### Principles & development of evidence-based practice

The term "Evidence-based medicine" was introduced in the medical literature in 1991 [26]. An original definition suggested the process was "an ability to assess the validity and importance of evidence before applying it to day-to-day clinical problems" [27,28]. The initial definition of evidence-based practice was within the context of medicine, where it is well recognised that many treatments do not work as hoped [29]. Since then, many professions allied to health and social care have embraced the advantages of an evidence-based approach to practice and learning [5-8,30]. Therefore we propose that the concept of evidence-based medicine be broadened to evidence-based practice to reflect the benefits of entire health care teams and organisations adopting a shared evidence-based approach. This emphasises the fact that evidence-based practitioners may share more attitudes in common with other evidence-based practitioners than with non evidence-based colleagues from their own profession who do not embrace an evidence-based paradigm.

EBP evolved from the application of clinical epidemiology and critical appraisal to explicit decision making within the clinician's daily practice, but this was only one part of the larger process of integration of evidence into practice. Initially there was a paucity of tools and programmes to help health professionals learn evidence-based practice. In response to this need, workshops based on those founded at McMaster by Sackett, Haynes, Guyatt and colleagues were set up around the world. During this period several textbooks on EBP were published accompanied by the development of on-line supportive materials.

The initial focus on critical appraisal led to debate on the practicality of the use of evidence within patient care. In particular, the unrealistic expectation that evidence should be tracked down and critically appraised for *all *knowledge gaps led to early recognition of practical limitations and disenfranchisement amongst some practitioners [31]. The growing awareness of the need for good evidence also led to awareness of the possible traps of rapid critical appraisal. For example problems, such as inadequate randomisation or publication bias, may cause a dramatic overestimation of therapeutic effectiveness [32]. In response, pre-searched, pre-appraised resources, such as the systematic reviews of the Cochrane Collaboration [33], the evidence synopses of *Clinical Evidence *[34] and secondary publications such as Evidence Based Medicine [35] have been developed [36], though these currently only cover a small proportion of clinical questions.

### Process of Evidence Based Practice

The five steps of EBP were first described in 1992 [37] and most steps have now been subjected to trials of teaching effectiveness (indicated by references)

1. Translation of uncertainty to an answerable question [38]

2. Systematic retrieval of best evidence available [39]

3. Critical appraisal of evidence for validity, clinical relevance, and applicability [40]

4. Application of results in practice [41]

5. Evaluation of performance [42]

This five-step model forms the basis for both clinical practice and teaching EBP, for as Rosenberg and Donald observed, "an immediate attraction of evidence-based medicine is that it integrates medical education with clinical practice" [43].

### Curricula outline of minimum standard educational requirements

Different practitioners at different levels of responsibility within evidence-based organisations will require different skills for EBP and different types of evidence. It is a minimum requirement that all practitioners understand the principles of EBP, implement evidence-based policies, and have a critical attitude to their own practice and to evidence. Without these skills and attitidues, health care professionals will find it difficult to provide 'best practice'. Teachers, commissioners, and those in positions of leadership will require appraisal skills that come with higher training and continued use [44].

The wider knowledge and use of these skills will help health professionals meet some of Hurd's list of desired educational outcomes [45] in being able to:

• distinguish evidence from propaganda (advertisement)

• probability from certainty

• data from assertions

• rational belief from superstitions

• science from folklore

### Curricula that outline the minimum standard educational requirements for practitioners

Evidence-based practitioners need additional skills to supplement traditional knowledge. Health care graduates should "be able to gain, assess, apply and integrate new knowledge and have the ability to adapt to changing circumstances throughout their professional life" [46]. Observational studies suggest that one way to 'future-proof' health care graduates, is to train them in the necessary skills to support life-long learning through the five-step model of EBM [47].

Learning has three components: knowledge, skills and attitudes. It is said that "attitudes are caught, not taught" [48]. Attitudes, such as comfort with managing uncertainty and reflective learning, provide the psychological framework in which evidence is appraised and applied, described by Sackett as "the *conscientious*, explicit and *judicious *use of current best evidence in making decisions about the care of individual patients" [49]. This presents a challenge, as EBP is rarely taught well [50] and is applied (and observed) irregularly at the point of patient contact [51] where professional attitudes are formed, and students learn to incorporate theory into practical skills for patient care. Patient involvement in decision making is part of the process of being an effective practitioner. The degree of involvement and the methods by which this is achieved will depend on the setting, the patients and the practitioner.

The curriculum framework for EBP should consider the importance of all steps shown in Table 1. Often courses focus on one of these elements, most commonly critical appraisal, but a balance of skills in each of the steps is needed to take a student from question through to application. Indeed, the most difficult step (sometimes dubbed "step 0") is to get students and colleagues to recognise and admit uncertainties. As Table 1 suggests, learning should be focused on educational outcome, which in turn needs to reflect the clinical setting. This practical orientation means that EBP teaching and assessment needs to consider the real-time setting of practice, and hence searching and appraisal need to be done in minutes rather than hours or days. Table 1 provides examples of established methods of teaching and assessment for each step, but further compilation, innovation, development, and testing are needed. Future research should be informed by the movement in best evidence medical education (BEME) [52].

**Table 1:** Description of evidence for aspects of Evidence-Based Practice teaching and assessment

**Educational outcome**	**Examples of methods of teaching**	**Examples of methods of assessment**
**Translation of uncertainty into an answerable question. **The student identifies knowledge gaps during the course of practice and asks foreground questions to fill these gaps, The student should ask focused questions that lead to effective search and appraisal strategies.	Presenting clinical scenarios or asking for students to share a problem encountered in clinical practice. Framing a focussed, answerable question in a structured format [38]. Several formats are taught: 3 part (patient-intervention-outcome), 4 part (patient-intervention/exposure-comparator-outcome), or 5 part (patient-intervention/exposure-comparator-outcome-time) questions.	The skills can be assessed by presenting a clinical scenario and asking the student to form a focussed, answerable question (included in the Fresno test) [53].
**Search for and retrieval of evidence. **The student can design and conduct a search strategy to answer questions. The strategy should be effective and comprehensive: likely to retrieve all relevant evidence. The student understands the strengths and weaknesses of the different sources of evidence.	Theoretical instruction backed by a supervised practical session with online connection [39]. A variety of databases should be shown such as Cochrane, MEDLINE, CINAHL, Evidence-Based Medicine, SumSearch, tripdatabase.com with the relative benefits discussed.	Computer based OSCE has been used to test the abilities of framing questions, searching, and retrieving appropriate evidence [54].
**Critical appraisal of evidence for validity and clinical importance. **The student can appraise the validity of a study. The appraisal will include: the suitability of the type of study to the type of question asked, the design of the study and sources of bias, the reliability of outcome measures chosen, and the suitability and robustness of the analysis employed. The student can appraise the importance of the outcomes and translate them into clinically meaningful summary statistics, such as number needed to treat (NNT).	This is probably the most widely taught skill [55]Examples include the Critical Appraisals Skills Program [56].	Tests for critical appraisal of validity include the Berlin Questionnaire [57] and the Fresno test.
**Application of appraised evidence to practice **The student can assess the relevance of the appraised evidence to the need that prompted the question. The student can explore the patient's values and the acceptability of the answer.	Examples include applying the identified evidence to the specific context that led to the quest for evidence. This requires exploration of the generalisability of the evidence to the specific scenario, and 'particularising' outcomes by adjusting for patient-specific risks[58].	Objective structured clinical examination involving clinical application and interaction with patient after reading supplied evidence [59].
**Evaluation of performance**. The student asks focussed questions, searches sources of evidence, appraises or uses pre-appraised evidence and applies these in practice. The student reflects on how well these activities are performed.	Role modelling by EBP teachers. The encouragement of adult learning styles. Journal clubs [60].	Use of a questionnaire to assess knowledge, attitude and behaviour [61].

### Recommendations

The term 'EBM' has evolved into a larger phenomenon, as increasing numbers of practitioners in various disciplines recognise the importance of evidence to inform all types of health care decisions. Furthermore, greater patient choice and complexity of care mean that many professionals practise as a team. In recognition of the importance of a united commitment to the principles of 'best practice', we propose that the term 'evidence-based practice' (EBP) be used to describe all aspects of this discipline.

To ensure that future health care users can be assured of receiving 'best practice' regardless of the type or location of the care received, we make the following recommendations for education:

1. The professions and their colleges should incorporate the necessary knowledge, skills and attitudes of EBP into their training and registration requirements.

2. Curricula to deliver these competencies should be grounded in the "five-step model" (Table 1).

3. Further research into the most effective and efficient methods for teaching each step should be fostered, and linked with ongoing systematic reviews on each step.

4. Core assessment tools for each of the steps should be developed, validated, and made freely available internationally.

5. Courses that claim to teach EBP should have effective methods for teaching and evaluating *all *components.

Evidence-Based Practice (EBP) requires that decisions about health care are based on the best available, current, valid and relevant evidence. These decisions should be made by those receiving care, informed by the tacit and explicit knowledge of those providing care, within the context of available resources.

Finally, EBP requires a health care infrastructure committed to best practice, and able to provide full and rapid access to electronic databases at the point of care delivery. We believe that without the skills and resources for all the relevant components of this framework, the practice of a health care professional, or a health care organisation, cannot be said to provide their users with evidence-based care.

## Summary

1. This consensus statement is from an international working group representing both organisations and individual teachers and developers of evidence-based practice.

2. Evidence-Based Practice (EBP) requires that decisions about health care are based on the best available, current, valid and relevant evidence. These decisions should be made by those receiving care, informed by the tacit and explicit knowledge of those providing care, within the context of available resources.

3. All health care professionals need to understand the principles of EBP, recognise it in action, implement evidence-based policies, and have a critical attitude to their own practice and to evidence. Without these skills professionals will find it difficult to provide 'best practice'.

4. The teaching of EBP should, as far as possible, be integrated into the clinical setting and routine care so that students not only learn the principles and skills, but learn how to incorporate these skills with their own life-long learning and patient care.

## Competing interests

All of the authors have received honoraria for teaching EBP FP is a consultant of Lilly Deutschland GmbH. The International Conferences of EBHC Teachers and Developers do not accept sponsorship from health technologies (including pharmaceutical) manufacturers.

## Authors contributions

MD, WS & PG wrote the original draft. AC, JM, KH, FP, AB & JO contributed to the concept and all revised drafts of the statement.

## Pre-publication history

The pre-publication history for this paper can be accessed here:


